# Robotic Distal Pancreatectomy for Adult Pancreatoblastoma with Tumor Extension to the Main Pancreatic Duct: A Case Report

**DOI:** 10.70352/scrj.cr.25-0037

**Published:** 2025-04-23

**Authors:** Hiroaki Sugita, Koji Amaya, Chihiro Kawata, Yasuhiro Nagaoka, Yoshitaka Iwaki, Yoji Nishida, Atsushi Hirose, Tomoya Tsukada, Takashi Nakamura, Masahiro Hada, Masaki Takeshita, Akemi Yoshikawa, Masahide Kaji

**Affiliations:** Department of Surgery, Toyama Prefectural Central Hospital, Toyama, Toyama, Japan

**Keywords:** pancreatoblastoma, adult, robotic surgery

## Abstract

**INTRODUCTION:**

Adult pancreatoblastoma (PB) is an extremely rare malignant pancreatic epithelial tumor. Although no standard treatment strategy has been established, complete resection is recommended for long-term survival. Here, we presented a case of an adult patient with PB who successfully underwent complete resection via robotic surgery. Furthermore, this is the first report of robotic surgery for PB, highlighting its novelty and potential clinical relevance.

**CASE PRESENTATION:**

A 40-year-old man presented with epigastric pain, and image examination revealed a well-defined tumor in the pancreatic tail extending into the main pancreatic duct (MPD) and reaching the pancreatic neck. With no evidence of distant metastases, surgery was planned following 2 courses of gemcitabine and S-1 chemotherapy for tumor shrinkage. There were no significant changes in the tumor’s extension after chemotherapy, but no new lesions appeared, and robotic distal pancreatectomy was performed. Intraoperative findings confirmed the tumor extension into the MPD just above the superior mesenteric vein (SMV). The pancreas was sharply divided at the right edge of the SMV, and a negative transection margin was obtained. The pancreatic stump was closed by suture. The postoperative course was uneventful, and the pathological diagnosis confirmed PB with MPD and inferior mesenteric vein invasion.

**CONCLUSIONS:**

We successfully resected an adult case of PB with tumor extension into the MPD via robotic surgery. Robotic surgery enabled precise pancreatic transection at the right edge of the SMV, ensuring a negative pancreatic transection margin in this case. In addition, robotic surgery contributed to the safe and secure suture closure of the pancreatic stump.

## Abbreviations

CT
: computed tomography
FDG
: fluorodeoxyglucose
GS
: gemcitabine and S-1
IMV
: inferior mesenteric vein
MPD
: main pancreatic duct
PB
: pancreatoblastoma
PDAC
: pancreatic ductal adenocarcinoma
SMV
: superior mesenteric vein

## INTRODUCTION

Pancreatoblastoma (PB) is a rare malignant epithelial tumor of the pancreas, comprising less than 1% of pancreatic tumors.^1)^ PB usually occurs in children, with a mean age of 4 years, and is extremely rare in adults.^2)^ Surgical resection has been reported to improve long-term survival.^3)^

We report a case of adult PB arising from pancreatic tail, with tumor extension into the main pancreatic duct (MPD) and thrombus in the inferior mesenteric vein (IMV).

Robotic distal pancreatectomy was successfully performed, and robotic surgery was employed for precise pancreatic transection and secure suture closure of the pancreatic transection stump.

Furthermore, this is the first report of robotic surgery for PB, highlighting its novelty and potential clinical relevance.

## CASE PRESENTATION

**Fig. 1:**
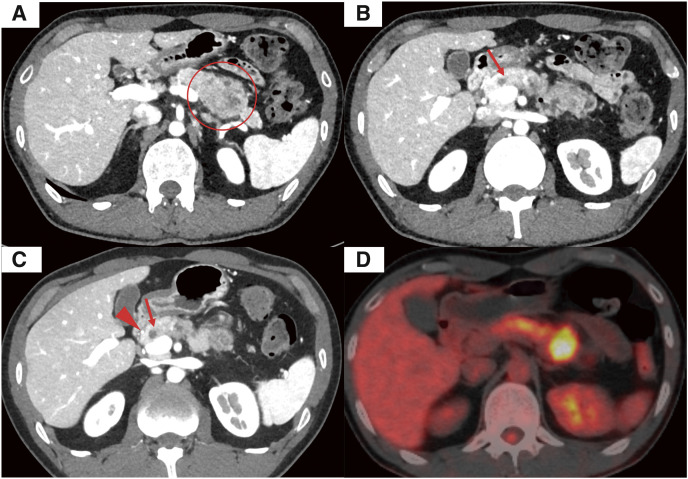
(**A**) Contrast-enhanced CT showing a well-defined tumor in the pancreatic tail (circle). (**B**) The tumor extended into the main pancreatic duct, with the progression reaching just above the superior mesenteric vein (arrow). (**C**) The leading edge of the tumor (arrow) was adjacent to the gastroduodenal artery (arrowhead). (**D**) FDG positron emission tomography-CT showing increased FDG uptake in the tumor, without lymph node involvement or distant metastases. CT, computed tomography; FDG, fluorodeoxyglucose

A 40-year-old male was referred to our institution for evaluation of epigastric pain. He had a history of childhood asthma. Contrast-enhanced computed tomography (CT) of the abdomen revealed a well-defined mass, approximately 30 mm in size, in the pancreatic tail (**Fig. 1A**). The mass was slightly enhanced in the arterial phase and extended into the MPD up to the pancreatic neck (**Fig. 1B**). The leading edge of the tumor was adjacent to the gastroduodenal artery (**Fig. 1C**), and the IMV was not visualized. Endoscopic ultrasound with fine-needle aspiration findings indicated that the tumor showed acinar differentiation and that acinar cell carcinoma should be the primary consideration; however, PB could not be ruled out due to similar findings. Fluorodeoxyglucose (FDG) positron emission tomography-CT showed increased FDG uptake in the tumor (standrd uptake value [SUV] max 8.53), without lymph node involvement or distant metastases (**Fig. 1D**). Serum levels of tumor markers such as carcinoembryonic antigen, carbohydrate antigen 19-9, and alpha-fetoprotein were within normal ranges. Considering the extensive involvement of the tumor, 2 courses of neoadjuvant chemotherapy with gemcitabine and S-1 (GS) were administered preoperatively in an attempt to reduce the extent of tumor growth. After that, contrast-enhanced CT showed no notable changes in tumor size or extent, but no new lesions were identified. Robotic distal pancreatectomy with splenectomy was planned for curative resection. Although the tumor was adjacent to the left adrenal gland, it had no obvious contact, so we planned a resection to expose and preserve the left adrenal gland.

**Fig. 2:**
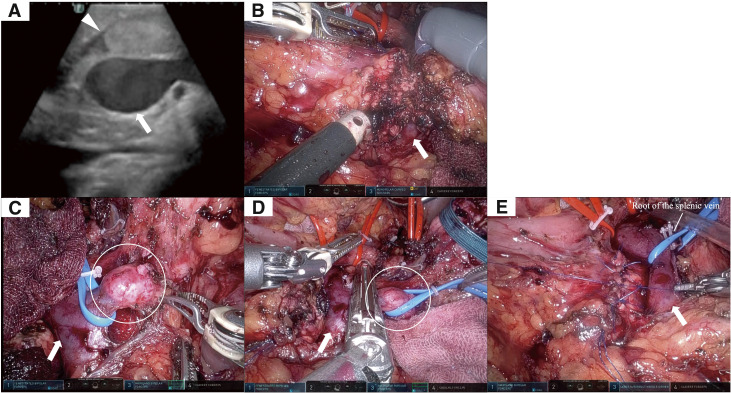
Intraoperative findings. Arrowhead: tumor; arrows: SMV. (**A**) Intraoperative ultrasonography revealed tumor extension into the main pancreatic duct just above the SMV. (**B**) Robotic surgery enabled sharp transection of the pancreas at the right edge of the SMV using monopolar curved scissors. (**C**) The IMV directly merged with the SMV, and a tumor thrombus (circle) was observed in the IMV. The tumor thrombus did not extend to the junction with the SMV. (**D**) The IMV was transected at its confluence with the SMV using a linear stapler (circle: tumor thrombus). (**E**) The pancreatic stump was precisely closed with sutures by the multi-joint function of robotic surgery. IMV, inferior mesenteric vein; SMV, superior mesenteric vein

Abdominal cytology was negative during the operation, and there were no non-curative factors. Intraoperative ultrasonography revealed tumor extension into the MPD just above the superior mesenteric vein (SMV) (**Fig. 2A**). Using monopolar curved scissors, the pancreas was sharply divided at the right edge of the SMV (**Fig. 2B**), and intraoperative frozen section analysis of the pancreatic stump revealed no malignant cells. In this case, the IMV directly merged with the SMV, but a tumor thrombus was observed in the IMV during surgery (**Fig. 2C**). The tumor thrombus did not extend to the junction with SMV, so the IMV was transected at the level of its junction using a linear stapler (**Fig. 2D**).

Anterior radical antegrade modular pancreatosplenectomy with lymphadenectomy was successfully performed without tumor exposure on the posterior dissection plane. After suture closure of the MPD stump, the pancreatic stump was securely sutured using multi-joint function of the robot (**Fig. 2E** and **Supplementary Video**). The operating time was 487 min, and the estimated blood loss was 94 mL. The patient was discharged on postoperative day 10 without any complications.

**Fig. 3:**
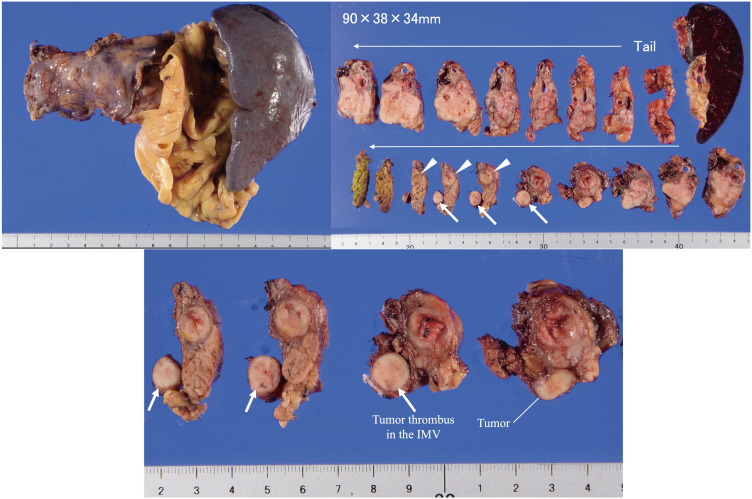
Macroscopically, the tumor was a well-circumscribed, lobe-shaped mass in the pancreatic tail, extending to the body and measuring 90 × 38 × 34 mm. It invaded the main pancreatic duct (arrowheads) and formed tumor thrombus in the IMV (arrows). The continuity between the tumor and the tumor thrombus in the IMV was confirmed. IMV, inferior mesenteric vein

**Fig. 4:**
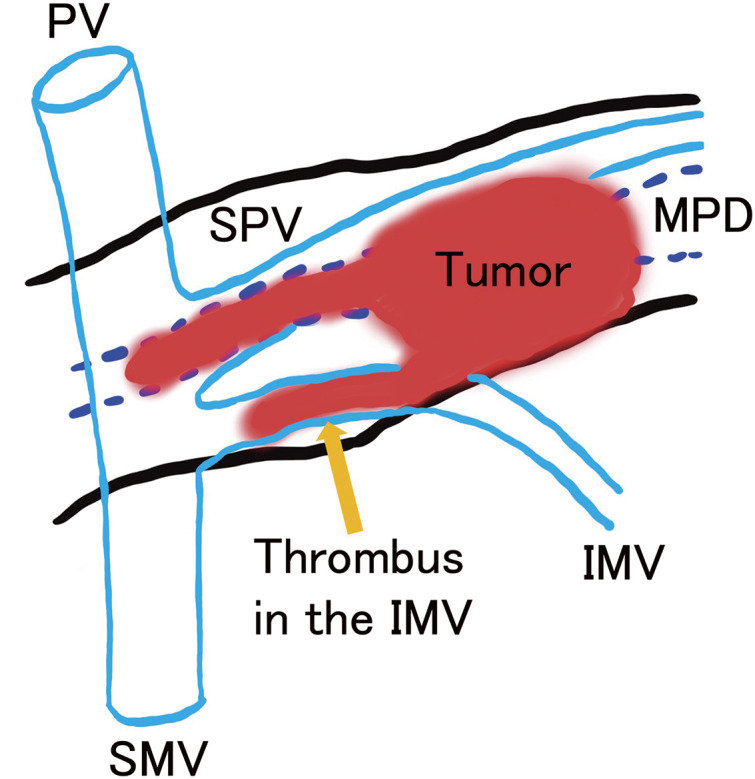
The scheme of the tumor extension and its anatomical location. The tumor extended into the MPD just above the SMV and invaded the IMV, forming a tumor thrombus. The tumor thrombus extended close to the confluence of the IMV with the SMV. IMV, inferior mesenteric vein; MPD, main pancreatic duct; PV, portal vein; SMV, superior mesenteric vein; SPV, splenic vein

**Fig. 5:**
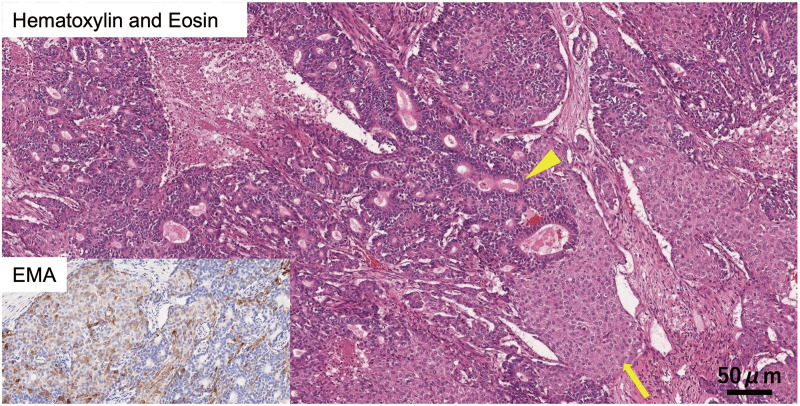
Pathological examination revealed that the tumor was composed of small glandular luminal or solid structures with acinar differentiation (arrowhead) and squamoid nests containing epithelioid cells with abundant eosinophilic cytoplasm (arrow) (hematoxylin and eosin staining, ×100). Immunohistochemically, the squamoid nests were positive for EMA. EMA, epithelial membrane antigen

Macroscopically, the tumor was a well-circumscribed, lobe-shaped mass in the pancreatic tail, extending to the body and measuring 90 × 38 × 34 mm. It invaded the MPD and caused a tumor thrombus in the IMV (**Fig. 3**). **Figure 4** shows a scheme of the tumor extension and its anatomical location. Histopathological examination revealed tumor cells with small, uniform, and round nuclei-forming nests with necrosis and fibrosis. The tumor displayed acinar differentiation, composed mainly of small glandular luminal or solid structures, with scattered squamoid nests containing epithelioid cells with abundant eosinophilic cytoplasm (**Fig. 5**). Immunohistochemical staining of the squamoid nests showed positivity for epithelial membrane antigen (**Fig. 5**) and Sal-like protein 4 (SALL4), with partial positivity for glypican-3. Some tumor cells showed neuroendocrine differentiation, with positive staining for synaptophysin and chromogranin A expression. Based on these pathological findings, a diagnosis of PB was confirmed. No lymph node metastases were observed. The patient is currently receiving adjuvant chemotherapy with S-1.

## DISCUSSION

PB in adults is extremely rare, with only 103 cases reported worldwide to date.^4)^ Among these, 78 patients underwent surgery, yet no cases of robotic surgery have been reported. This report describes the first case of successful resection of adult PB using robotic surgery.

PB is a relatively slow-growing tumor with no characteristic symptoms, leading to delayed presentation and substantial tumor size by the time of diagnosis.^5)^ Abdominal pain and awareness of an abdominal mass are often the main complaints,^6)^ as observed in this case. Approximately 25% of adult PB patients present with metastases at initial diagnosis.^7)^ PB can invade adjacent organs or tissues, with the liver being the most common site of metastasis.^8)^ In this case, the tumor invaded the IMV and formed a tumor thrombus, confirming its susceptibility to transportal liver metastasis.

Complete surgical resection is the most recommended treatment for adult patients with PB, offering the possibility of long-term survival.^1,4)^ Resectability depends on factors such as the presence or absence of distant metastases, tumor size, location, and extent of invasion. In this case, although the tumor was primarily localized to the pancreatic tail, its extension into the MPD and toward the pancreatic neck necessitated precise pancreatic transection at the right edge of the SMV to ensure negative pancreatic margins. In laparoscopic distal pancreatectomy, pancreatic transection is widely performed using a linear stapler with prolonged peri-firing compression to prevent postoperative pancreatic fistula.^9)^ However, this approach can be challenging when pancreatic transection is performed near the right edge of SMV owing to spatial limitations, and suturing after sharp pancreatic transection is difficult using the conventional laparoscopic approach. In this case, robotic surgery provided precise pancreatic transection along the right edge of the SMV and enabled reliable suturing of the pancreatic stump. The multi-joint function of robotic system facilitated secure and accurate closure. Additionally, regarding pancreatic transection, ligation of the resected side of the pancreas was not performed to avoid tumor crushing in this case. However, to prevent tumor spread, some form of intervention may be desirable. In cases where the tumor extends into the MPD and is close to the pancreatic transection line, careful measures should be taken to avoid tumor crushing. These include loose ligation or gentle compression using atraumatic forceps or intestinal clips to minimize the risk of tumor damage.

Regarding chemotherapy for PB, the PLADO regimen, a combination of cisplatin and doxorubicin, has been reported to be effective in pediatric cases, demonstrating a response rate of 73%.^10)^ In adult PD, in addition to the PLADO regimen, 5-FU-based chemotherapy, such as FOLFIRINOX (5-fluorocrail + leucovorin + irinotecan + oxaliplatin), is also frequently used.^4)^ As for postoperative adjuvant chemotherapy, the PLADO regimen is often utilized in pediatric cases.^10)^ However, there are currently no established criteria for the optimal duration or selection of target cases. In our case, since the patient was young, neoadjuvant chemotherapy was administered with the aim of tumor reduction to avoid total pancreatectomy. Although its efficacy was uncertain, GS therapy was performed following the protocol for pancreatic ductal adenocarcinoma (PDAC) due to insurance coverage constraints in Japan. In the current case, preoperative GS was judged to have had some effect on tumor control, as no tumor progression was observed. Therefore, considering the risk of recurrence of liver metastasis due to the IMV tumor thrombus, we initiated adjuvant chemotherapy with S-1, similar to the approach used in PDAC. For adult PB, due to the rarity of the disease, there is no established evidence regarding adjuvant chemotherapy,^11)^ and further accumulation and analysis of cases are needed in the future.

Considering the relatively young age of the patient and the need for adjuvant chemotherapy, robotic surgery proved advantageous in terms of both curability and minimal invasiveness.

## CONCLUSIONS

In conclusion, adult PB is a rare pancreatic malignant tumor, and the establishment of an appropriate treatment strategy, along with the accumulation of case reports, is crucial for understanding and managing this disease. When accompanied by MPD extension, as in this case, the precise pancreatic transection and suture techniques of robotic surgery are useful for optimal resection.

## SUPPLEMENTARY MATERIALS

Supplementary Video Intraoperative ultrasonography revealed tumor extension to just above the SMV. The pancreas was encircled at the left side of the gastroduodenal artery and transected sharply using monopolar curved scissors just along the right edge of the SMV. The main pancreatic duct was closed by Z suture. Intraoperative findings revealed tumor thrombosis in the IMV, and the IMV was transected at the confluence with the SMV using a linear stapler. After completion of distal pancreatectomy with splenectomy, the pancreatic stump was securely sutured using multi-joint function of the robot.

## ACKNOWLEDGMENTS

We would like to thank Editage (www.editage.com) for English language editing.

## DECLARATIONS

### Funding

The authors did not receive any specific grant from funding agencies in the public, commercial, or not-for-profit sectors.

### Authors’ contributions

All authors agree with the content of the final manuscript.

HS contributed to drafting manuscript.

KA, CK, YN, YI, YN, AH, TT, TN, MH, MT, AY, and MK contributed to revising the manuscript.

### Availability of data and materials

All data written in this article are available from the first author upon reasonable request.

### Ethics approval and consent to participate

This work does not require ethical considerations or approval. Informed consent to participate in this study was obtained from the patient.

### Consent for publication

Informed consent for publication of this case report was obtained from the patient.

### Competing interests

The authors declare that they have no competing interests.

## References

[1] Mahdi M Abu Alnasr M Almehman BA A rare case of pancreatoblastoma in a pediatric patient. Cureus 2020; 12: e6779. 32015939 10.7759/cureus.6779 PMC6986467

[2] Klimstra DS. Nonductal neoplasms of the pancreas. Mod Pathol 2007; 20: S94–112. 17486055 10.1038/modpathol.3800686

[3] Salman B Brat G Yoon YS The diagnosis and surgical treatment of pancreatoblastoma in adults: a case series and review of the literature. J Gastrointest Surg 2013; 17: 2153–61. 24081396 10.1007/s11605-013-2294-2

[4] Yuan J Guo Y Li Y. Diagnosis, treatment, and prognosis of adult pancreatoblastoma. Cancer Med 2024; 13: e70132. 39162366 10.1002/cam4.70132 PMC11334167

[5] Li J Peng C Fan X Adult pancreatoblastoma: a case report. J Int Med Res 2021; 49: 3000605211039565. 34461770 10.1177/03000605211039565 PMC8414932

[6] Omiyale AO. Clinicopathological review of pancreatoblastoma in adults. Gland Surg 2015; 4: 322–8. 26312218 10.3978/j.issn.2227-684X.2015.04.05 PMC4523633

[7] Zouros E Manatakis DK Delis SG Adult pancreatoblastoma: a case report and review of the literature. Oncol Lett 2015; 9: 2293–8. 26137059 10.3892/ol.2015.3001 PMC4467354

[8] Zhang D Tang N Liu Y Pancreatoblastoma in an adult. Indian J Pathol Microbiol 2015; 58: 93–5. 25673604 10.4103/0377-4929.151199

[9] Nakamura M Ueda J Kohno H Prolonged peri-firing compression with a linear stapler prevents pancreatic fistula in laparoscopic distal pancreatectomy. Surg Endosc 2011; 25: 867–71. 20730447 10.1007/s00464-010-1285-6

[10] Bien E Godzinski J Dall’Igna P Pancreatoblastoma: a report from the European cooperative study group for paediatric rare tumours (EXPeRT). Eur J Cancer 2011; 47: 2347–52. 21696948 10.1016/j.ejca.2011.05.022

[11] Nunes G Coelho H Patita M Pancreatoblastoma: an unusual diagnosis in an adult patient. Clin J Gastroenterol 2018; 11: 161–6. 29285688 10.1007/s12328-017-0812-6

